# Réponse spectaculaire au valproate de sodium d’une chorée de Sydenham récurrente

**DOI:** 10.11604/pamj.2017.27.212.11383

**Published:** 2017-07-20

**Authors:** Siham Bouchal, Ouarda Ouali, Mouhamed Faouzi Belahsen

**Affiliations:** 1 Service de Neurologie, CHU Hassan II, Fès, Maroc

**Keywords:** Sydenham´s chorea, recurrent chorea, sodium valproate, Sydenham's chorea, recurrent chorea, sodium valproate

## Abstract

La chorée du Sydenham est la première cause de chorée acquise dans le tiers monde. Nous rapportons le cas d'une Chorée récurrente traitée avec succès par le valproate du sodium. Mlle A.C âgée de 16 ans ayant comme antécédents des angines à répétition et un épisode de mouvement choréique il y a 2 ans, pour lequel elle a été mise sous Halopéridol et sous prévention du rhumatisme articulaire aigu. La patiente a interrompue le traitement et quelques mois plus tard elle a présenté le même tableau neurologique. L'IRM cérébrale et l'échographie transthoracique étaient normales. La prévention par Extencilline a été réinstaurée ainsi que l'halopéridol sans aucune amélioration, d'où la mise en route sous valproate de sodium. La réponse était spectaculaire après 2 mois de traitement sans récidive après 3ans de recul. Le traitement de la chorée du Sydenham était les neuroleptiques. Des études récentes préconisent d'autres molécules efficaces et mieux tolérées.

## Abstract (en)

Sydenham's chorea is the most common acquired cause of chorea in the third world. We report a case of recurrent chorea successfully treated with sodium valproate. Miss A.C, aged 16, with a history of recurrent angina reported an episode of choreic movements 2 years before requiring treatment with haloperidol and prevention of rheumatic fever. The patient interrupted her treatment with occurrence of a relapse a few months later. Brain MRI and transthoracic ultrasound were normal. Preventive treatment with extencilline and haloperidol was restarted without any improvement, hence the treatment with sodium valproate. The patient responded very well after 2 months of treatment, without recurrence at 3 years' follow-up. The treatment of Sydenham's chorea was based on neuroleptics. Recent studies advocate the use of other more effective and better tolerated molecules.

## Introduction

Les mouvements anormaux posent souvent des difficultés diagnostiques et thérapeutiques. La chorée dérive du mot latin « choreus » qui veut dire « danse ». Elle se caractérise par des mouvements involontaires irréguliers rapides souvent généralisés et pouvant intéressés même la face. Elle est d´étiologie variable et la chorée du Sydenham représente la première cause de chorée acquise chez l'enfant dans le tiers monde. Le traitement classique de la chorée de sydenham associé une antibioprophylaxie du rhumatisme articulaire aigue (RAA) et l´Halopéridol. Nous rapportons le cas d'une Chorée récurrente traitée avec succès par le Valproate de sodium [1,2].

## Patient et observation

**Figure 1:**
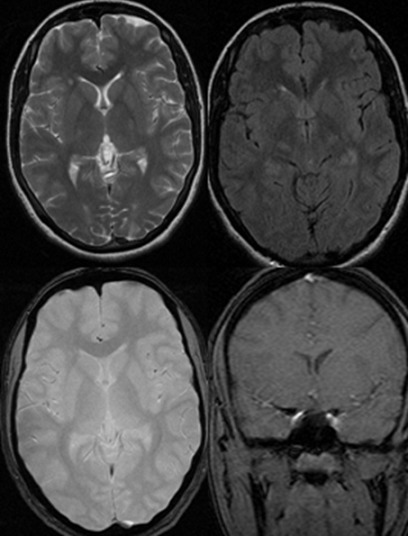
IRM cérébrale coupe axiale T2, FLAIR, T2* et coupe coronale après injection de gadolinium: normale

Mlle A.C âgée de 16 ans ayant comme antécédents des angines à répétition et un épisode de mouvement choréique avec un syndrome inflammatoire biologique il y a 2 ans, pour lequel elle a été mise sous Halopéridol et sous prévention secondaire du rhumatisme articulaire aigu (RAA). Sous traitement, l´évolution a été marquée par la disparition complète des symptômes après quelques mois. La patiente a interrompu l'antibioprophylaxie du RAA après 3 mois. Une année après le premier épisode, elle a présenté une récidive des mouvements choréiques généralisés. L'examen clinique initial a révélé des mouvements choréiques intéressant les 4 membres et la face et gênant la marche. L'examen de la gorge était normal. L´IRM cérébrale était normale (Figure 1). Le bilan biologique (ASLO; NFS; VS) et le bilan immunologique (Anticorps antinucléaire et Anticorps anti-DNA) étaient normaux. L'électrocardiogramme et l'échographie transthoracique n'ont rien révélé de particulier. Le diagnostic de chorée de Sydenham récurrente en dehors d'une poussée de RAA a été posé. La prévention secondaire par Benzathine Benzylpénicilline a été réinstaurée ainsi que l´Halopéridol à dose sans aucune amélioration clinique. La patiente a été mise sous Valproate de sodium à dose de 1000mg/jour progressivement. La réponse au Valproate de sodium était spectaculaire, marquée par la régression quasi complète après 2 mois de traitement. Avec un recul de 3ans après le second épisode, il n'y a eu aucune récidive. Le valproate de sodium a été arrêté après une année de stabilité clinique, alors que la Benzathine Benzylpénicilline est maintenue jusqu´a l´âge de 21 ans.

## Discussion

La chorée de Sydenhm (CS), appelée également « danse de saint Guy », constitue une des manifestations neurologiques du rhumatisme articulaire (RAA) et fait partie des critères majeurs de JONES [1,2].Ce rhumatisme, qui est en voie d'éradication dans les pays industrialisés en raison de l'utilisation des antibiotiques et de l'amélioration de la qualité de vie, reste encore fréquent dans les pays du tiers monde. La physiopathologie de cette chorée reste encore mal élucidée mais l'hypothèse la plus admise est celle d'une réaction auto-immune croisée post-infectieuse par mimétisme moléculaire entre les épitopes de la membrane du streptocoque et l'isoganglioside GM1 des neurones des noyaux gris de la base. C'est en 1976 que Mr Husby et ses collaborateurs ont identifié les anticorps anti-noyaux gris centraux (ABGC) dans les sérums de 50% à 90 % de patients atteint de formes aigues de la chorée de Sydenham. D'autres auteurs suggèrent l'hypothèse d'une vascularite intéressant les noyaux gris secondaire à l'infection [1,3,4]. La CS est fréquente chez le sexe féminin avec un âge moyen entre 5-15 ans. Elle survient quelques semaines après une infection de la sphère ORL à streptocoque β-hémolytique du groupe A. Elle se caractérise par des mouvements involontaires qui touchent tous les muscles à l'exception des muscles oculaires. La chorée est souvent généralisée bien qu'une hémichorée soit décrite. Le diagnostic d'une chorée post-streptococcique repose sur un faisceau d'arguments épidémiologiques, cliniques et biologiques couplés à la normalité de l'imagerie cérébrale [4]. La CS est généralement monophasique. Cependant, quelques récidives, ont été rapportées dans la littérature et le délai de récurrence est très variable, de quelques mois à plusieurs années [5]. L'hypothèse d'un taux élevé des ASLO ou la mauvaise observance de l'antibiothérapie prophylactique sont discutées comme facteur de risque de récurrence, mais aucun paramètre clinique ou biologique ne permet de le prédire [5]. La récurrence peut être concomitante à une poussée de rhumatisme articulaire aigue ou isolée. Ainsi, il est recommandé de réaliser un bilan pour éliminer d'autres étiologies avant de retenir le diagnostic de chorée du Sydenham récurrente [5].Chez notre patiente la récidive est survenant après l´arrêt de l'antibiothérapie prophylactique et la chorée dans le 2eme épisode était isolé sans autre élément du RAA. Le traitement symptomatique classique de CS était l'Halopéridol. Des essais cliniques récents ont prouvé l'efficacité du Valproate de sodium (20-25mg/Kg par jour) avec moins d'effets secondaires que l'halopéridol. Certains le proposent ainsi que la Carbamazépine en première intention [6,7]. Des petites séries ont comparé le Valproate de sodium et la Carbamazépine (10-15mg/kg/jr) et elles n'ont pas montré de différence significative aussi bien sur l'efficacité, sur la tolérance que sur le risque de récurrence [6,8].

## Conclusion

La CS peut être monophasique ou récurrente. La récidive peut être intégrer dans une poussée du RAA ou isolée. Son traitement repose sur les neuroleptiques. Cependant, des essais cliniques récents ont révélé l'efficacité de la Valproate de Sodium, de la Carbamazépine ou d´autres molécules qui sont aussi efficaces et mieux tolérées. Le moyen thérapeutique le plus efficace de la CS reste la prévention par un traitement précoce et adapté des infections au streptocoque béta-hémolytique du groupe A.

## Conflits d’intérêts

Les auteurs ne déclarent aucun conflit d'intérêts.
